# Converting data into knowledge with RCA methodology improved for inverters fault analysis

**DOI:** 10.1016/j.heliyon.2022.e10094

**Published:** 2022-08-12

**Authors:** Ricardo Manuel Arias Velásquez, Jennifer Vanessa Mejía Lara

**Affiliations:** a Universidad Privada Peruano Alemana, Peru; b Universidad Nacional de San Agustín de Arequipa, Peru; c Pontificia Universidad Católica del Perú, Peru

**Keywords:** Failure mode, Inverters, Knowledge management, Solar plant

## Abstract

In the last years, the knowledge management methodology increased the perspective and deeply analysis in the energy evaluation, with great emphasis in the training of the maintenance teams and early detection of failure modes; these inefficiencies detection is associated to patterns recognition with expert systems. Several energy brands, utilities, universities, and design companies investigated about this problem with limits in the integration between maintenance team knowledge and the degradation of the energy equipment. Therefore, our findings are a new approach of the root cause analysis (RCA) improved with the knowledge management perspective, associated to the failure mode analysis for 164 inverters in photo-voltaic solar plant by using twenty-one failures modes; by incorporate the graph theory called Erdös–Rényi graphs with a quantitative methodology and qualitative evaluation with the knowledge management method in the root cause analysis; the dataset evaluated has 120,561 signals associated to 3,014,025 patterns, during the period from 2018 to 2021 in a PV solar plant. In this new root cause analysis method, the knowledge management is analyzed as a complement for the solution for sudden failure modes and early degradation.

## 1 Introduction

Since 2020, the decarbonization challenge is the main goal for one hundred eighteen countries around the world, especially these targets for solar technology requires high reliability, energy availability and low lost production, in countries as Denmark, Sweden, China, United Kingdom, Thailand, and other countries [1]. For instance, a special case is Nigeria, it has an aggressive strategy for the solar projects with ranking of all the technologies, strong regulations and key industrial [2]. However, this global growth won’t be sustainable without improving the analysis of the failure modes of the PV solar plants; hence, this problem contributes to the difficulty of predicting its production in the short term, added to the randomness of the climate such as the presence of clouds and the increase in wind. Companies and government should investigate new ways to detect failures and plan maintenance actions, to avoid sudden failures in the systems [3]. Firstly, the step done in 2020 created an innovative framework with the evaluation of Pruned graph; in particular, it demonstrates the pruned causal map after using the several conditions (topologies of the power plant). In this direction, graphs theory brings us an interesting approach for understand the failures modes in a solar photo-voltaic (PV) plant [3].

Previously, in 1991 Nonaka Takeuchi incorporates the knowledge management chain into the decision-making process; furthermore, during the 2021 in Ref. [4], the authors proposed a knowledge exchange through several systems in industry companies, by collaboration between teams, in the energy chain “system operators, independent power producers and transmission service providers” [4]. A main problem of the energy companies is to retain specialized engineers, to spread the knowledge. As revealed in Ref. [5], companies have 25% of the faults, in Latin America, associated to human error during the maintenance or energy projects [5]. Consequently, this fault rate had a boost during 2020 and 2021, associated to the constraints of the pandemic COVID-19 and the consequence digitalization stage in the industrial process, in this way, the causal mapping with a qualitative research method called the Gioia. This method tried to recognize faults with lower number of energy workers in the industry, especially due to the pandemic, capacity restrictions and massive infections; thus, implementation of the knowledge management, as an innovation booster, to reduce the failure rate; but the relation between variables hasn’t been recognized.

Overall, with Refs. [5] and [6] a restriction is the maintenance team collaboration and the complexity level according to the energy technology and identification of the context [6]. Notable, the social networks are used in the analysis of organizational context with the information of the organizational flux and comparison of knowledge between areas by using “exponential random graph modeling” [7] for the evaluation.

As an example, in the last decade, some approaches have considered the influence of the spatial analysis and the time parameter [7, 8]; for example: In 2012, the bottom-up graphic Gaussian model (GGM) with neighborhood similarity [22], it has process with spatial analysis and influence of several failures' modes but low precision with more than one failure mode at the same time. On the other hand, the limits of the knowledge management application were a linear contribution in the root cause analysis [5], with expert systems. However, the knowledge doesn’t increase according to new evidence, with an additional constraint by the computational time in the complex systems. Therefore, the reduction of the variables helps to reduce the computational time, for instance, the principal components and factorial analysis are the techniques used to reduce the time and variables. About the root cause analysis process, in the international standards as IEC 62740 [19], it is developed in five steps, but it is recommended for linear process.

Finally, in 2020, a theoretical application with artificial datasets in the training model, also with a spatial-temporal analysis has been developed a theory without a real case study as a limitation; evidently, the team and human behaviors are considered as ideal, without effects in the root cause analysis [21]. As a complementary, in 2021, the JRP-DBSCA method with recurrence theory for fault diagnosis and root cause analysis has a nonlinear description, but the temporal analysis is not considered [23]. Therefore, a root cause analysis with spatial and temporal analysis in complex systems haven’t been developed, yet.

### 1.1 Motivation

The root cause analysis is a complex analysis in energy systems as Photo-voltaic power plants, based on the international standards as IEC 62740 [19] for RCA and IEC 60812 [20]. In particular, these standards are not helping in the spatial – temporal analysis for failure modes analysis, and in the complex fault propagation mechanism; as instance the converter DC/AC control system or in a complete PV solar plant are not linear behavior with several failure modes. Usually, the tree decision model used in the standard has biases in management and even worse a high probability to obtain only a perspective in the equipment without the procedures, policy, team skills and other people factor in the analysis. In 2020, a new theory is proposed with minimization problem through the inference based metric, based on sequential state switching and artificial anomaly association with precision of 90.6%, recall 96.7% with synthetic data [21], in this research, we propose an improvement in the theory and the implementation of a case study with a photo-voltaic plant and company with data from the 500,000 solar panel and 775,785,600 data sets for solar irradiance and active power during the period 2019 to 2021, the results has a contribution of precision of 99.2% recall 99.6% and F-measurement of 99.6%.

The knowledge contribution of this research article are as follows: The proposal for the root cause analysis improved with the knowledge management approach by using the “Erdos-Renyi model” [8, 9]; indeed, this analysis allows to incorporate the maintenance team interactions and several failure modes. It provides a visual description of the failures and the knowledge nodes. An accuracy of 98.3% compared with theoretical report compared with the same database on Ref. [3]; with several failures' mode at the same time by compared the algorithm Random Committee and Logistic Model Tree with Erdos-Renyi model with spatial-temporal analysis for RCA associated to graph-based support vector machine; the information from the real case study has obtained an accuracy of 99.2%. We reduced the graph according to inverters and conversion units instead of a complete solar photovoltaic plant. We have detected the evolution of the failure’s modes and the corrective maintenance evaluation though the years. In 2019, a similar approach with “graph-based support vector machine model has been developed for the electroencephalography signal” [10] with successful application. The contribution of Ref. [10] produces several “shell and fixed according to specific values”; of the resulting graph are principally strong minded by the “bifurcation number of the original Cayley tree” [11].

This research article is composed in five sections, as follows: Section 2 describes the methodology of the root cause analysis improved with knowledge management techniques. Section 3 introduces the case study with a real photovoltaic solar plant with discussion associated to inverter failures, results detailed, and the root cause analysis. Section 4 develops the discussion about the knowledge contribution to the international standards and results. Finally, the last section is associated to the conclusions and future works.

## 2 Methodology and data

### 2.1 Comparison with the state-of-the-art methods

Currently, according the state of art there are two main perspectives in the root cause analysis methodology:

• A general method for linear process
• A data-driven approach with nonlinear analysis.

The description of the limits, benefits and applications of the solar technology are indicated in Table 1.

**Table 1:** Limits and benefits in the actual methods.

Methods	Benefits	Limits	Complex systems
GGM with neighborhood similarity 2012 [22].	Process with spatial analysis and influence of several failures' modes at the same time.	Low precision with more than one failure mode.	It doesn’t consider the time degradation and the identification.
Knowledge management perspective (2017), [5].	An approach for linear approach with expert systems, associated to previous experience.	It has not considered detection of faults. The knowledge doesn’t increase according new evidence. Main sensors have considered in the analysis according Principal Components methodology.	It has not considered complex problems, more than 2 failure modes in the same time.
IEC 62740 2015 [19] with the complementary IEC 60812 2018.08 [20]	A simple process with five steps for linear events.	The detection is not considered in the scope. RCA is required, the focus event(s) to be analyzed is described and an appropriate team appointed for the analysis, with human error influence.	It doesn’t consider complex problems with spatial and temporal influence.
Sequential state switching and artificial anomaly association, 2020 [21]	A theoretical application with artificial datasets in the training model. An analysis with spatial-temporal analysis.	It doesn’t consider the human factor and industrial environments and constraints.	It considers ideal teams without maintenance evaluation.
JRP-DBSCA method: Recurrence theory for fault diagnosis and root cause analysis of nonlinear and unstable multivariate [23].	Interpretation of multiple and complex pattern according to clustering methods.	It allows to determine complex failure’s modes in the spatial analysis with high accuracy with causality and transfer entropy analysis in five and three faults with 86% and 93% respectively of accuracy.	It doesn’t consider the time evaluation and degradation.

In solar technology, the influence of the data-driven based on the available signal in the inverters – converter DC/AC, panels, transformers, strings, and control systems in the spatial and temporal analysis should be considered, besides, the maintenance team and organization.

The main inefficiencies detected in the state of art for the solar technology, and scope of this paper are twenty-eight [3], as follows:

- Irradiance measure failure.
- Pyranometer condensation.
- Converter DC/AC failure.
- Gateway or signal converter fault.
- Electronic card’s fault.
- Ageing of panels, inverter, cables and transformers.
- IGBT failure.
- Fan or source fan failure in the converter DC/AC or Cabin unit.
- Filters for harmonics.
- Humidity in the inverters.
- Dust in the inverter or soiling over the panels.
- Snow or ice over the panels.
- Limitation of the inverter caused by control failure or protection.
- Electrical failure (signal or power system).
- Thru fault.
- Electrical test (corrective or preventive maintenance).
- Noise level.
- High temperature in the panel, inverter, transformers or cables.
- Active parts with low insulation.
- Cooling system fault.
- Tracker fault or unavailability.
- Operational data unavailability.
- Load constraints.
- Design issues.
- Communication or intermittence.
- Clouds over the solar power plant.
- Shadow effect, S-effect over the panels.
- Ageing or degradation of the panels: Yellowing, crack, among others.

### 2.2 Methods

In Table 2, the evaluation of main application of graph theory are the following:

- Dimensions analysis evaluates to graph theory and Fractal with support vector machine (SVM) application, it allows to reduce the dimension and obtain with SVM the accuracy of 95.6% with the evaluation of Fractal; the limitations are the small dataset for a fully validate the Graph theory with a Fractal improvement [10].
- In the Graph theory-based standardized matrix modelling method, the limitation is the heat transfer analysis according to the topology in the accuracy for non-linear process. The matrix-based modeling approach applies a redefined thermal resistance for heat transfer analysis to obtain unified linear constraints of thermal system with an accuracy of 91.1%.
- Finally, the graph theory is applied to clustering analysis, in application to renewable energy, the optimization performance is higher than 0.8%–1.05%, compared with the traditional methods.

**Table 2:** Comparison with the methodology used with graph theory according systematic review.

Methodology	Comparison and accuracy applied in energy application
Graph theory with a Fractal improvement-based sine cosine driven support vector machine (2019) [10].	It requires a dimensional reduction and a fractal dimension calculation with a selection of optimal support vector machine parameter with an accuracy of 95.6%.
Graph theory-based standardized matrix modelling (2022) [25].	It requires a hierarchical model for the application, digraph and arrangement of nodes with its definitions and edges, with an optimization of 91.1%.
Clustering via a Sparsified wake digraph (2022) [26].	Graphical representation from spatial and temporal analysis and representation of renewable energy as wind farms. Specially the representation of turbulence from 9% to 15%; the optimization performance gain rate falls to only 0.87% and 1.05%,
Erdos-Renyi models as study of topology (2020) [13]	In Ref. [13], the mathematical analysis of the Erdos-Renyi model, it allows scaling approach to the study of topological indexes. In this case, for prediction and representation of temporally analysis, the Erdős–Rényi results agreed well with the real data [27].

**Figure 1:**
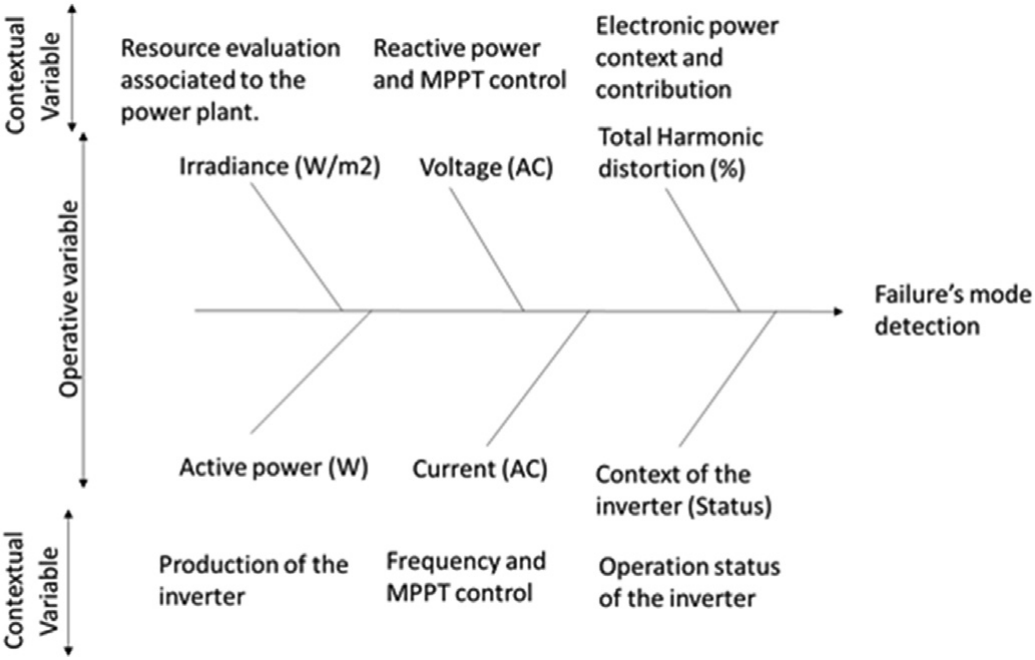
Quantitative design.

The research article considers an inductive process associated to a specific case study to the generalization of the theory. The design is a quantitative and qualitative research: In the quantitative design: The independent variables are indicated in Figure 1, as follows:

- The resource evaluation associated to the power plant with the information from the irradiance installed in the sun trackers.
- The reactive power and MPPT control associate to the voltage in ac from the DC/AC converter,
- the power electronic context and contribution of the DC/AC converter, filters and IGBT, with the Total Harmonic Distortion (THD).
- The production of the inverter is associated to the active power in Watts,
- The frequency and MPPT control, associated to the current from the DC/AC converter.
- Finally, the context of the inverter is associated to the status. About the dependent variable, is the failure’s mode detection in the inverter, in Figure 1.

**Figure 2:**
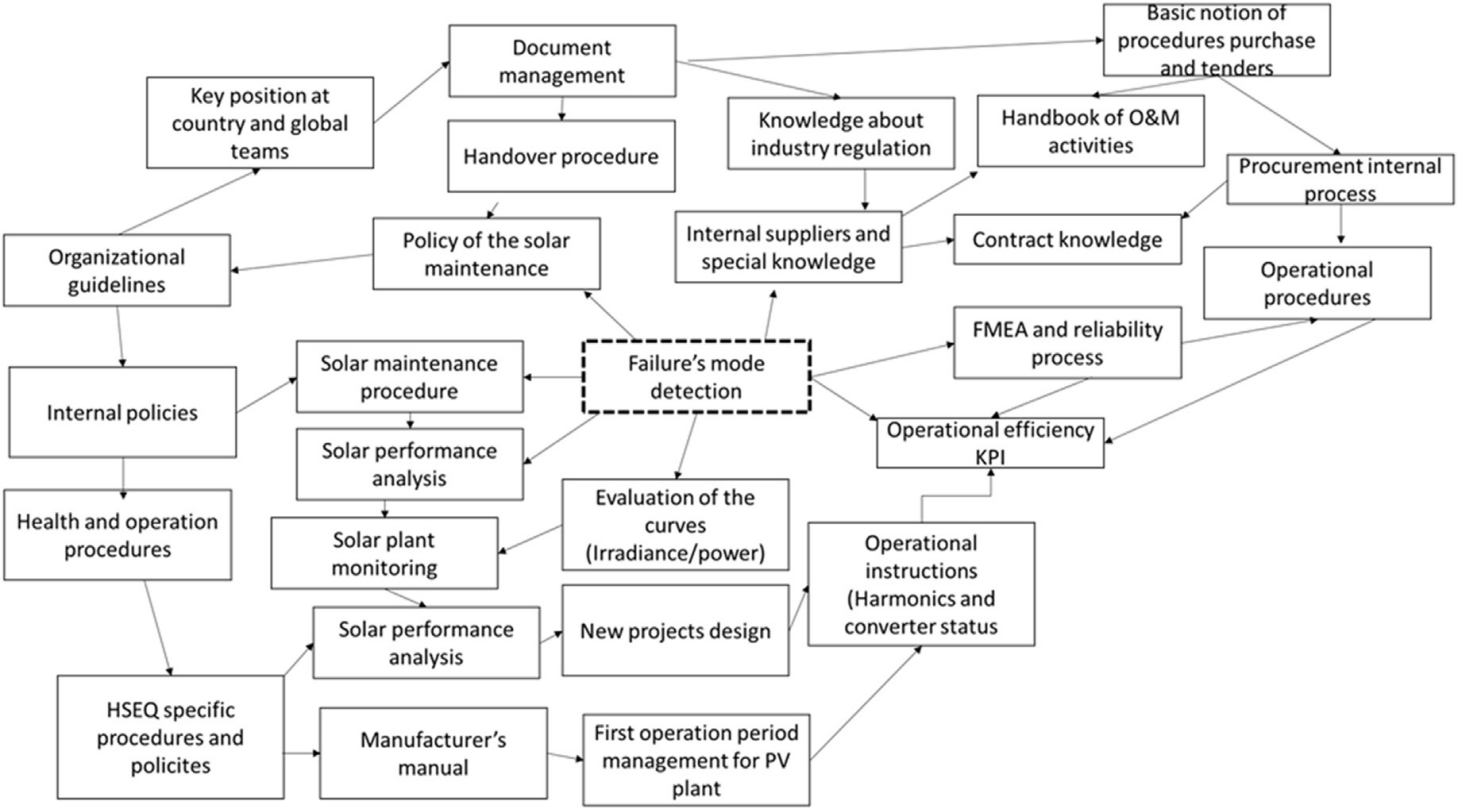
Qualitative design for the evaluation of the failure’s mode detection in solar plants.

In Figure 2, the main factors associated to the failure’s mode detection with direct influence are seven: i) Policy of the solar maintenance, ii) solar maintenance procedure, iii) solar performance analysis, iv) evaluation of the curves, v) operational efficiency KPI, vi) Failure mode and effect analysis (FMEA) and reliability process, vii) internal suppliers and special knowledge. The details are the following:

• Policy of the solar maintenance has an influence in the organizational guidelines, and it receives influence of the handover procedure (process for new power plants or equipment from the engineering and construction to operation and maintenance.
• The solar maintenance has influence of the organizational guidelines thought the internal policies; it allows to improve the solar performance analysis.
• The evaluation of the curves for the irradiance and active power sets, it has a direct influence in the solar monitoring and solar performance analysis.
• The operational efficiency and KPI has changed due to FMEA and reliability process, and it evaluates the operational procedures.
• The internal suppliers and special knowledge have influence in the contract knowledge and handbook (web service) of O&M activities.

For the evaluation of the knowledge management, a survey is associated to qualitative design with 31 factors for the failure’s mode detection, described in Table 3.

**Table 3:** Factors evaluated with the Cronbach’s alpha and correlation.

Variables	Description of the	Cronbach’s alpha	Total correlation
S1	Policy of the solar maintenance	0.92	0.63
S2	Key positions at country and global teams	0.91	0.64
S3	Documentary management	0.91	0.55
S4	Handbook of O&M Activities from new projects and O&M	0.91	0.76
S5	Internal market and supplier’s knowledge	0.91	0.58
S6	Solar Plant Maintenance procedure	0.91	0.52
S7	Organizational guidelines	0.91	0.59
S8	Knowledge about industry regulation	0.91	0.61
S9	Project execution procedure	0.91	0.72
S10	Solar Plant Monitoring	0.91	0.61
S11	Contract knowledge	0.91	0.62
S12	Evaluation of the curves (irradiance/power)	0.91	0.8
S13	Operational instruction	0.9	0.55
S14	Health and operation procedures	0.9	0.53
S15	Internal Policies	0.9	0.65
S16	Manufacturer’s manual	0.9	0.66
S17	First Operation period management for PV Plant	0.9	0.72
S18	Plant Maintenance and Monitoring of Solar Power Plants	0.9	0.59
S19	Legislation and regulations for import and export	0.89	0.6
S20	New projects design	0.89	0.75
S21	Operational Procedures	0.89	0.54
S22	HSEQ specific procedures & policies	0.88	0.72
S23	FMEA and reliability processes	0.88	0.78
S24	Hand over procedure	0.88	0.59
S25	Reference Power Curve for Solar Plant	0.88	0.58
S26	Procurement General procedures: Procedures for contracting international freight, customs clearance, licenses and authorizations.	0.88	0.61
S27	Solar Power Curves Handbook	0.88	0.69
S28	Solar Performance Analysis	0.86	0.66
S29	Procurement Internal procedures: Direct procurement to supplier, contractual additive.	0.86	0.56
S30	Operational efficiency and KPI	0.86	0.62
S31	Basic notion of procedures purchases and Tenders	0.84	0.59

### 2.3 Root cause methodology applied to inverter analysis

According the root cause methodology, the failure event is defined with the “Disconnection of one or more inverter units” and “Partial or total loss of the power of the PV solar plant”, associated to one conversion unit (one to four DC/AC converters) or several conversion units, respectively.

For the analysis, the three protections present in the DC/AC converters are the voltage, current and frequency, it is evaluated in real time. Each protection device depends directly on the electrical variable for which it is configured and indirectly on the other electrical variables due to the relationship between them and the electrical grid.

**Figure 3:**
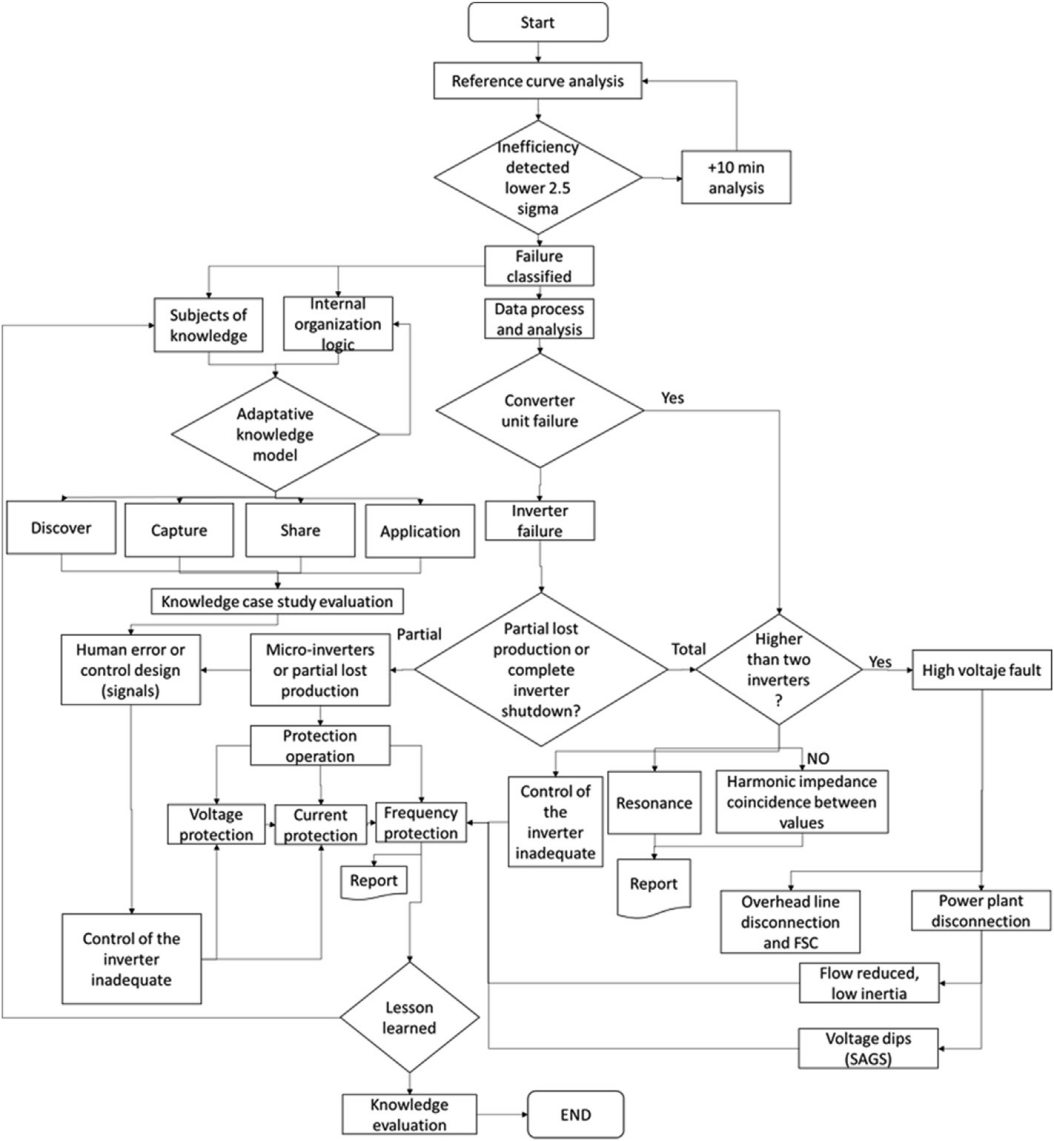
Methodology proposed for the root cause analysis for inverters in PV solar plant.
• The second stage is the fault classified stage is indicated in the inverter according the status of the inverter and the information from the PV solar plant with twenty-one failures modes and actions, as follows: Extension of planned maintenance, ageing, automatic action of the inverter, corrective planned maintenance, corrective un planned maintenance, extreme temperature in the inverter, faults in the inverter and strings, grid fault in the high voltage grid, lack of spare parts, natural disaster, not classified due to communication problems, predictive checks and maintenance, proactive maintenance, soiling, technical inefficiency problems for trackers, physical inspections in inverters, shadow effect, calibration of the inverter, outage, punch list for the project, technical interference due to changes in the trackers.

We have applied the “problem of change point detection (CPD)” [12]; it used to identify the evolution of faults in systems in classical time series data according the distribution of a particular value, the function or variable. In order to define the connection between nodes, we used the graph theory [9] associated to the curve inefficiency analysis and the limits with the 2.5 sigma for the quality evaluation, from the connections of each inefficiency point. “The psychological data in the theoretical model” has been used for human error studies, for the root cause analysis [9]. The evaluation of the curves for the inefficiency compared to the probability “p” of Erdös–Rényi graphs with the regime of mostly isolated vertices [13]. However, it requires the multipartite digraph defined in the AH model considered the graph theory. The methodology has been composed from three stages:

• Reference curve analysis: In Figure 3, the methodology started with the evaluation of the power curve analysis: active power (kW) vs irradiance (w/m^2^), each inverter has a manufacturer curve [3]. In this case, it has considered the evaluation 2.5 sigma for the lowest quality limit with the 10 min analysis.

**Figure 4:**
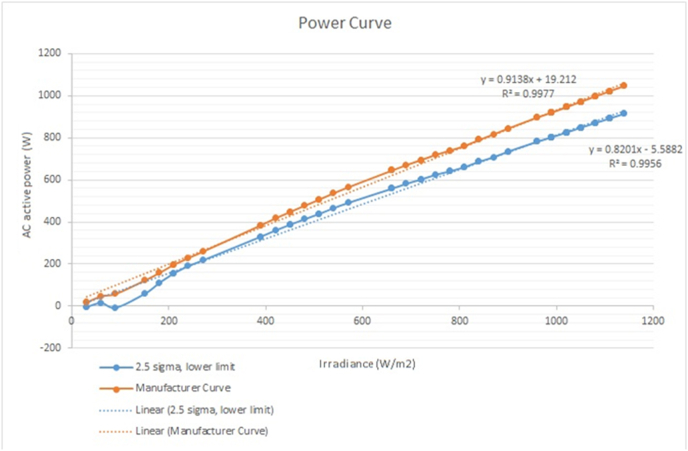
Manufacturer curve and quality limit for inefficiency.

It is evaluated according low value, below the 2.5 sigma curve limit, in Figure 4.

• Data processing and analysis: The data analysis considers the graphs feature for each inverter V and time T for a locality region $G_{T}\left(V\right)$ and the sub graph $G_{T}$, in Eq. (1).

(1) $$G_{T}\left(V\right)=\delta \left(N_{1}\left[V;G_{T}\right];G_{T}\right)$$

In Eq. (1), $G_{T}\left(V\right)$ is induced with the neighborhood of each vertex V.

The vertex is estimated according the size of the region $G_{T}\left(V\right)$ and the β with Ө called “vertex-dependent normalizing parameter estimates” [15], then ${\left(size\left(G_{T}\left(V\right)\right)\right)}^{\prime }$; it means the normalized activity estimate in the regular action, in Eq. (2), with the evaluation through the years.

According to the measurement evaluated in PV solar plant, it was evidenced that in most of the cases analyzed, the overcurrent protection disconnects the inverters, due to the increase in this variable.

According to the analyzes carried out for the sudden losses in production in a short period, called (Runbacks). During this event, the inverter current increases in the Runback, however, these current increases were not severe enough to generate a disconnection of the inverters. Therefore, it is suggested that there is an additional cause for the current growth, unusual current peaks appear, reaching 5 kA.

In these tests an abnormal behavior of the Power Frequency control P(f), this control was found when the inverter recovers after a voltage drop. Nevertheless, for voltage drops (dips) are defined as a momentary increase in RMS voltage of 10% or more above specify (rated) equipment voltage for a period of 1/2 cycle to 1 min, as defined in the IEC 61000-4-30 (SAGS) [14], greater than 50%, which generates current increases, followed by a disconnection of the inverters already turn a loss in the plant’s production.

Additionally, a problem was observed in the control system related to an injection of reactive power with the wrong sign, which further contributes to the current increase mentioned above.

The inadequate operation of the inverter control system occurs when there is fluctuation in variables such as voltage and frequency, originating from events in the National Interconnected Electric System (NIES), with some problems in DC (fuses [28], cables and panels) and AC side (filters, inverters and transformers) [3].

Finally, with all the elements described above, the cause analysis method, in Figure 3.

(2) $${\left(size\left(G_{T}\left(V\right)\right)\right)}^{\prime }=size\left(G_{T}\left(V\right)\right)-\frac{\beta \left(V\right)}{\theta \left(V\right)}$$

(3) $$T_{t}^{E}={\left(size\left(G_{T}\left(V\right)\right)\right)}^{\prime }$$

The large value of Eq. (3) is associated to extreme communication activity.

It allows to estimate messages from ω(V) to $T_{t}^{E}$, according graph theory in Ref. [13], Eq. (4) evaluates the larges values of $T_{t}^{E}$ with communication change.

(4) $$T_{t}^{E}=\underset{V}{\sum }I\left\{\arg\;max_{k}\;\omega \left(V\right)\neq arc\;max_{k}\;\omega_{t-1}\left(V\right)\right\}$$

**Figure 5:**
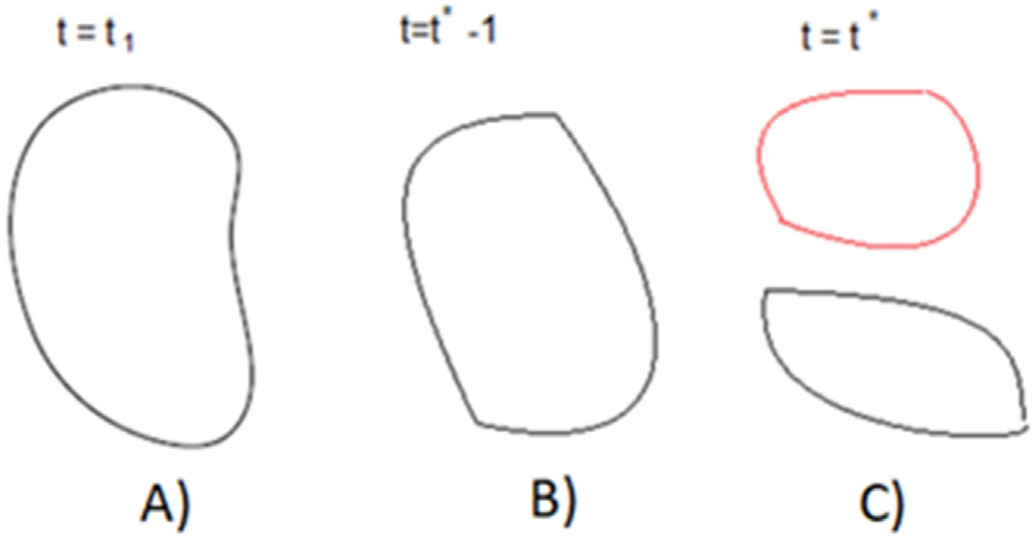
Anomaly for time series of random graphs, from t1 through t∗-1 in the period analyzed. A) Times series in the period T1. B) The previous period with anomaly in the time series of random graphs. C) The next period of the time series through t∗1.

This target allows to detect an evolution of the failure modes associated to inverters in PV solar, according to the inefficiency points (under the 2.5 sigma) during a specific period, in Figure 5.

**Figure 6:**
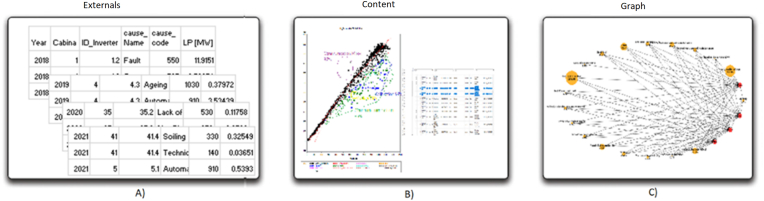
The external information is provided from the inverter. A) External information for year, cabinet unit, inverter number, cause name, cause code, and lost production associated. B) Evaluation of the cure of the inverter. C) graph modelling for the inverter and the lost production.

In general, it allows to incorporate the graph theory. The evolution of the failures and the results of the corrective maintenance, in order to compare the recent past. “The null hypothesis, then, is some form of time-based similarity-no probabilistic behavior changes in terms of either graph”, in Figure 6.

The “vertex-dependent normalization” [13] $T_{t}^{E}$, and critical values determined by recent past with a cluster collection, developed in Ref. [16]; with training messages used in this research, according Eq. (5). In this equation, the mutual information from context and content is the vectors associated to corpus “M”, in the data set for an instantaneous period, it is called “x”, therefore, $f_{x,w}$ is the $\frac{f_{x,w}}{N}$, with N is the total number of points associated to irradiance and active power.

(5) $$M_{x,w}=log\left(\frac{f_{x,w}}{\left(\sum_{w}\;f_{N,w}+\sum_{M}\;f_{M,w}\right)\sum_{w}\;f_{x,w}}\right)$$

With this approach, for the individual statistic for $T_{t}^{E}$ and $T_{t}^{C}$ is associated to local solutions for PV solar plants, it could be described in Eq. (6).

(6) $$T_{t}^{E\&C}=max_{V}g{\left(size\left(G_{T}\left(V\right)\right)\right)}^{\prime },\;\left\|\left(\theta {\left(V\right)}^{\prime }\right)-\left(\theta_{t-1}{\left(V\right)}^{\prime }\right)\right\|$$

### 2.4 Implementation process

The implementation process is developed in Figure 3, with six steps:

- Step 1: Reference curve analysis: A calibration of the system with the manufacturer curve according the converter DC/AC. Besides, to create the limit of 2.5 sigma.
- Step 2: An evaluation of the knowledge management characteristics, according 31 factors in Table 3, the focus is the solar technology and the maintenance team knowledge and company.
- Step 3: All the signals of the conversion unit (CU), panels and substations are linked to the database system: Current, voltage, frequency, control and settings parameters, converter DC/AC status, temperatures (inverter, transformers, panels, CU), irradiance, meteorological stations (clear index, rain, environmental temperature, global horizontal irradiance (GHI), global tilted irradiance (GTI), pressure), oil temperature.
- Steps 4: The training model detects the failures' mode online and with the data process and analysis.
- Step 5: With the manufacturer curve and the limits, the calculation of the loss production is detected. A partial failure or more than 2 converters DC/AC or high voltage equipment are detected with timestamp data. The clustering analysis and graphs methods determinate the normal operation in the spatial and temporal analysis, the detection of low limits and initiates new evaluation.
- Step 6: The evaluation of the degradation and fault with control and protection analysis generates the report and lesson learned in order to increase the training model. Finally, the knowledge evaluation and root cause analysis. It determines one or more failures' mode with the training of the maintenance teams and detection of the failures' mode and inefficiencies with patterns recognition. In this step, the interpretation of the maintenance team is crucial and the evaluation of the 31 factors allow to increase the knowledge, with new lesson learned and the continuous evaluation.

## 3 Case study

**Figure 7:**
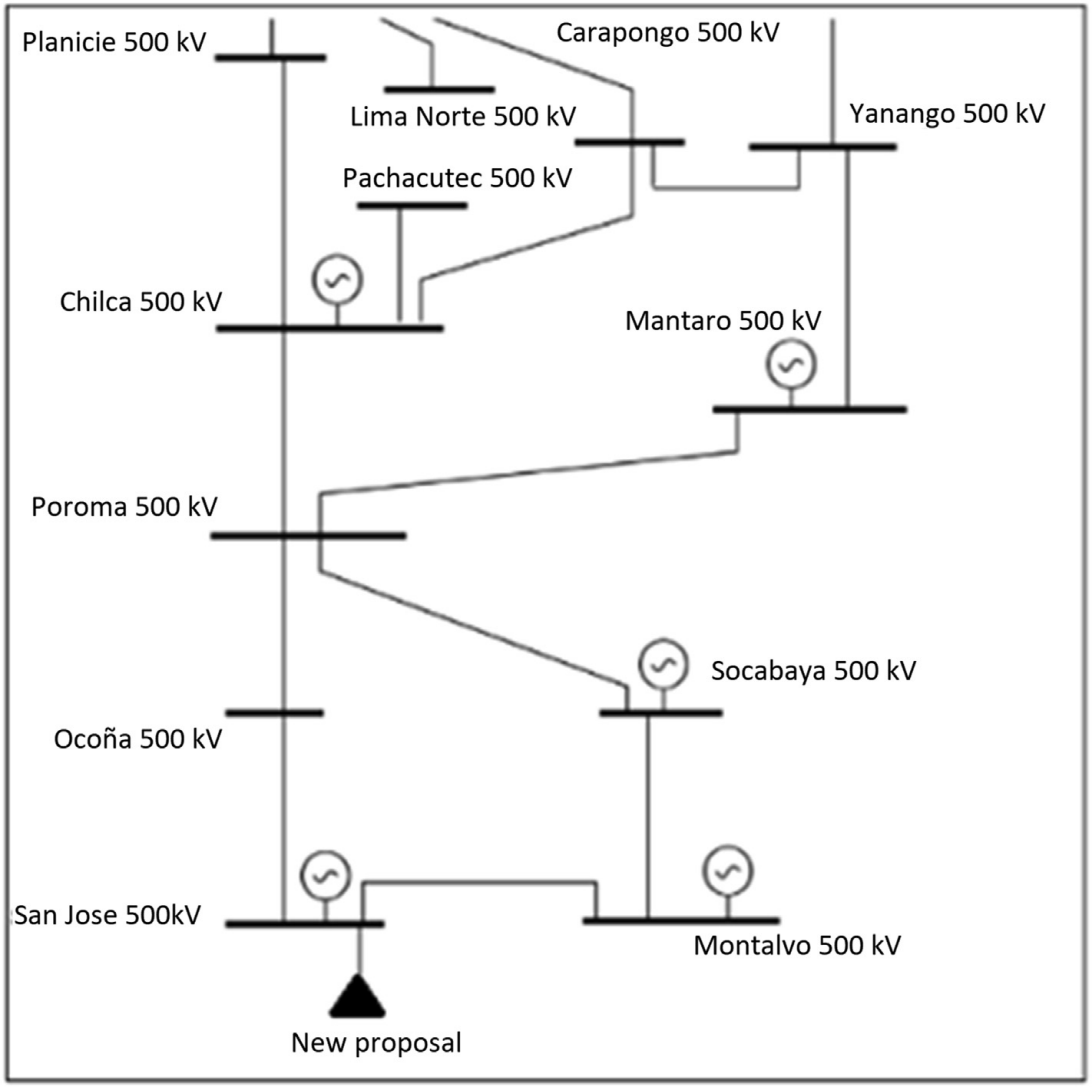
Location of the case study.

The evaluation of the Peruvian Photovoltaic solar plant in Peru, located in industrial environments. The location is in the San Jose substation, in a 500 kV grid ring. In this stage, it has three renewable non-conventional plants in 220 kV and two in 138 kV, in Figure 7.

**Figure 8:**
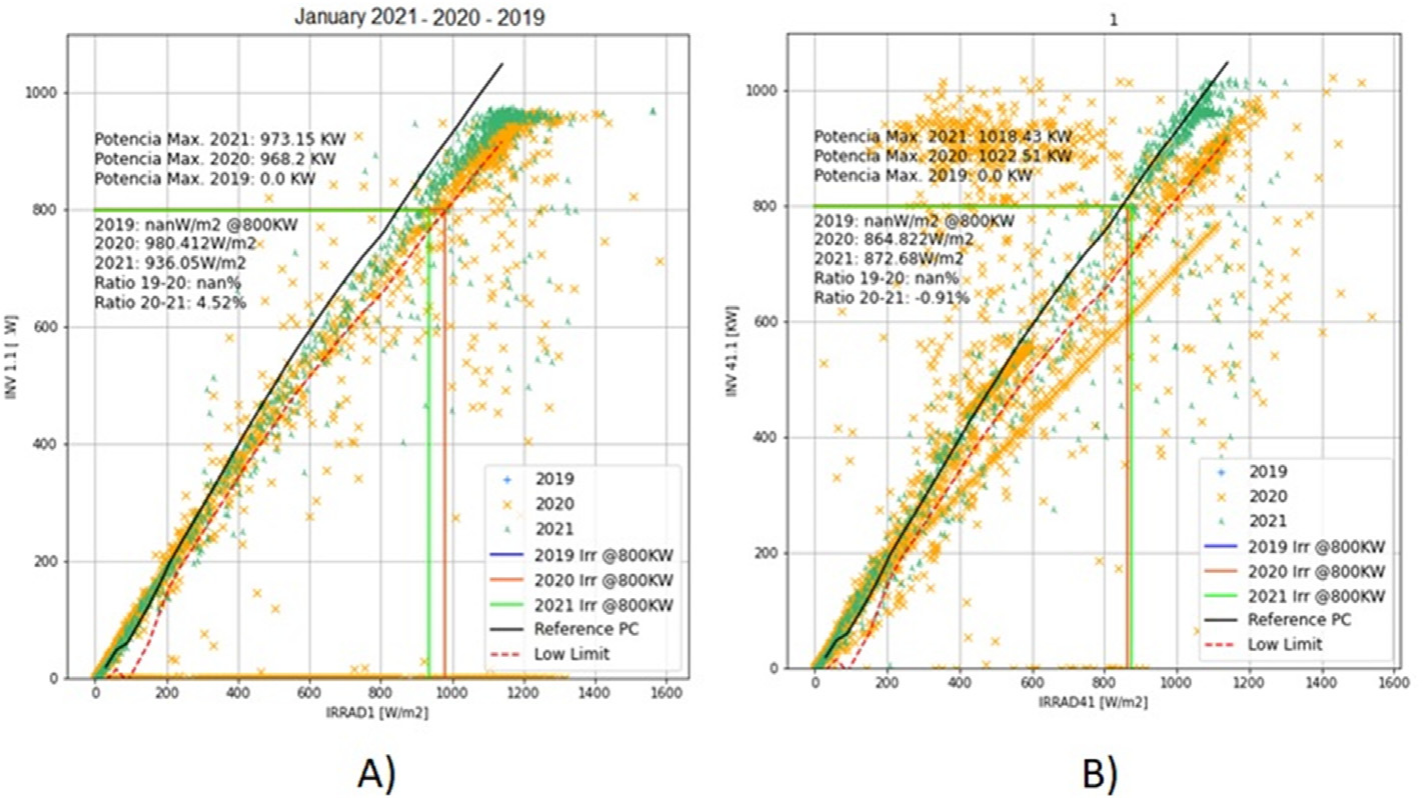
Evaluation of manufacturer curve (indicated as Reference power Curve (PC), 2.5 sigma (low quality limit) and the production active power vs irradiance per inverter. A) Inverter 1 for January 2019, 2020 and 2021. B) Inverter 164 for January 2019, 2020 and 2021.

The case study is developed in 164 inverters for solar panels systems, in 1700 m above sea level, with 500,000 solar panels, in the case study. The information for the classification is 775,785,600 data sets for solar irradiance and active power during the period 2019 to 2021; in this case, the evaluation is per month. In Figure 8, it has incorporated the data analysis of the inverter 1 and inverter 164.

The evolution of the failure’s modes inverter through the years, it has considered the information of the inverter 1.4 (inverter 4) and the inverter 41.4 (inverter 164), as follows:

Inverter 4:

• 2018: During the first year, the main failures are alternate current (AC) filter fault, technical inspections, and corrective planned maintenance.
• 2019: Later, during the second year, the failures modes have been increased as automatic action, corrective planned maintenance, extreme temperature, ac filter fault, predictive checks and maintenance, proactive maintenance, shadow effect and ageing.
• 2020: A big impact with external failures as ac filter fault has affected the inverter, and automatic actions, corrective planned mai-ntenance.
• 2021: Finally, during the fourth year, the main impact is the corrective maintenance, automatic action, ac filter fault, soiling, among other.

**Figure 9:**
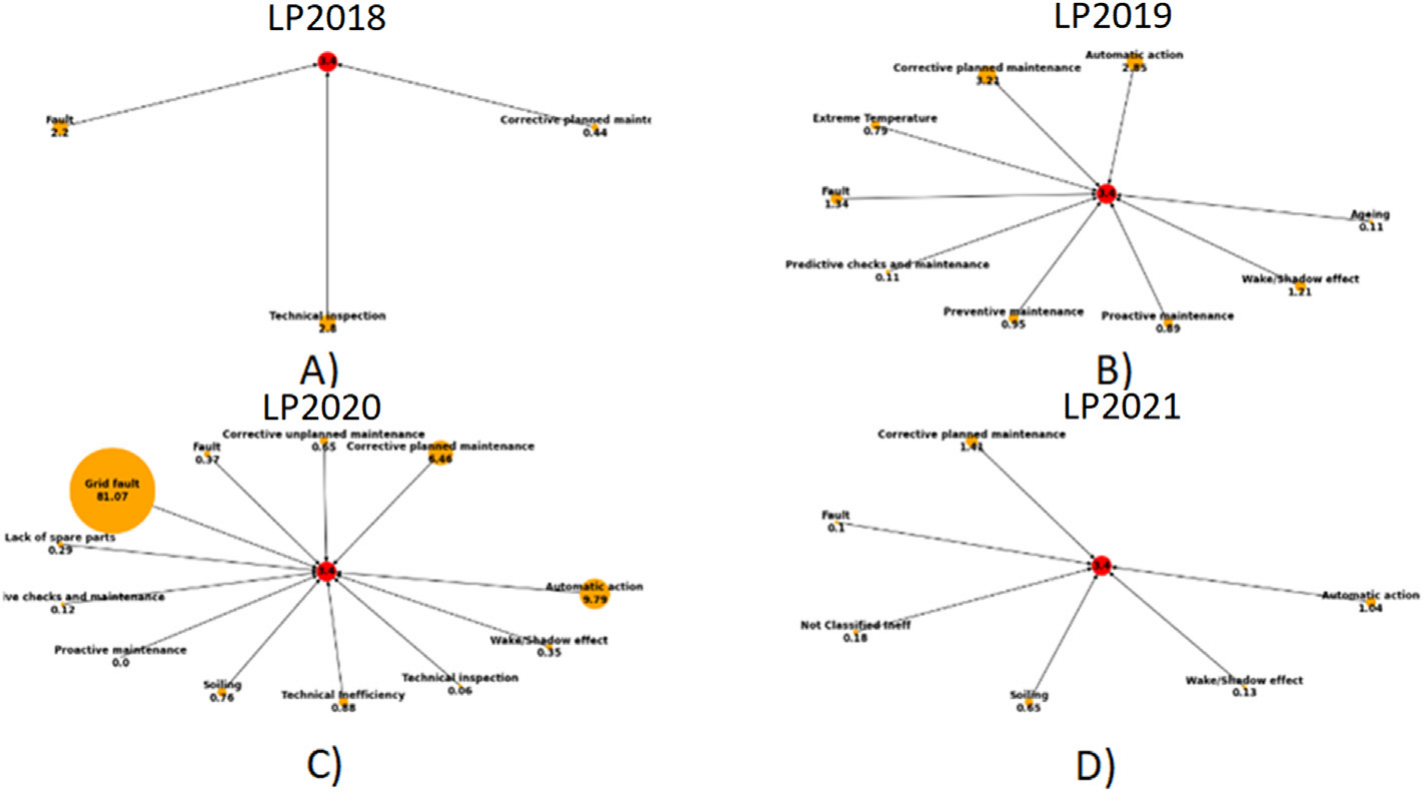
Context application is applied to obtain the time series graph G(V), for the inverters 161, 162, 163 and 164, for the spatial – temporal analysis. A) year 2018. B) year 2019. C) year 2020. D) year 2021.

Inverter 164:

• 2018: During the first year the main failures are punch list, ac filter fault, technical inspections, and corrective planned maintenance.
• 2019: Later, during the second year, the failures modes have been increased as follows: Natural disaster, automatic action, corrective planned maintenance, extreme temperature, ac filter fault predictive checks and maintenance, proactive maintenance, shadow effect and ageing.
• 2020: A big impact with external failures as automatic actions, grid fault (ac filter fault), corrective planned maintenance, and so on.
• 2021: Finally, during the fourth year, the main impact is the automatic action, corrective maintenance, ac filter fault, soiling, among other; in Figure 9.

Figure 9 illustrates the lost production caused by the failure mode, it increased during 2018–2020, however in the 2021, it has been reduced in the 2021 by the root cause analysis.

**Figure 10:**
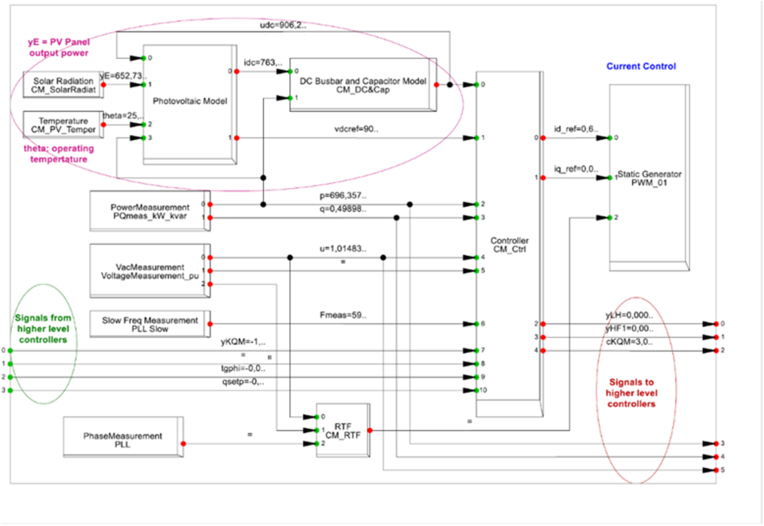
Description of the inverter and the independent values.

In Figure 10, the inverter model is used, the photovoltaic model is used from the solar radiation (Irradiance) and the temperature in the panel with the feedback from the DC current busbar input and the power ac. Besides, in green colour, the information of the signals from higher level controllers from the grid, as frequency, voltage and active power. The phase locked loop (LPP), allows to synchronise the inverter and the grid, associated to the PWM static inverter control.

According the hypothesis, it introduces a matrix evaluated with 120,561 data sets associated to 3,014,025 blocks, during 2018–2021, Eq. (7).

(7) n=|V|=120,561

### 3.1 Impedance analysis as function of frequency

The common failure mode during all the years are the ac filter fault, the root cause analysis according Figure 9, the irradiance, voltage, current, active power and operation status are considered in the analysis with the IEEE 519 standard and modelling with the DC/AC model in Figure 10.

**Figure 11:**
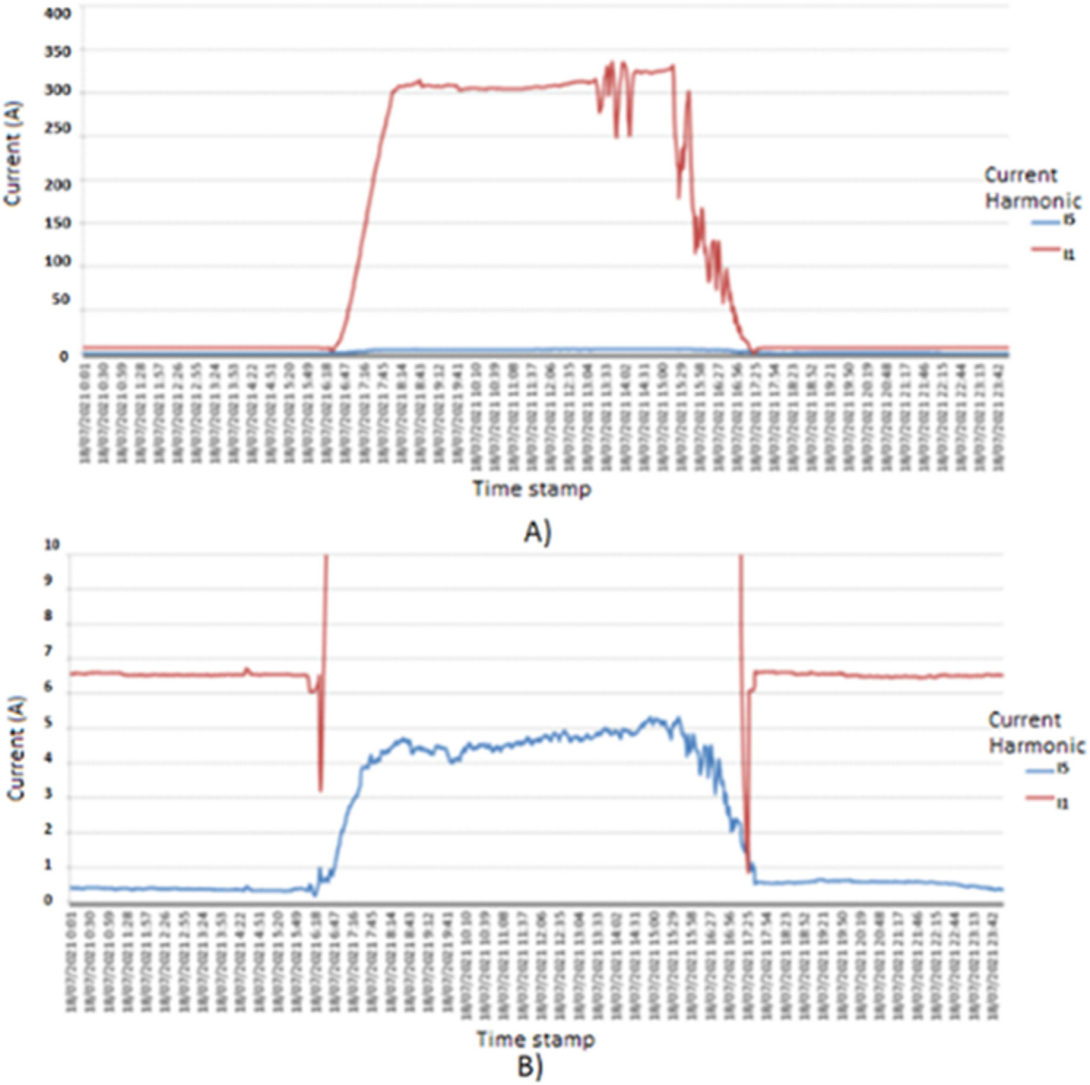
Reduction of the fundamental current. A) Current evaluation for fundamental current and fifth harmonic; normal condition. B) Current evaluation for fundamental current and fifth harmonic; failure contribution.

The next step detects variations in the impedance (Z (ω)) associated with the topological changes that occurred in the electrical grid during the event, according 3 different stages, with two factors:

• Current or voltage distorsion in the AC filters: It increases the current or voltage, from the results obtained in the simulation, it was observed that during the sequence of events in the grid, it coincided with the failure of AC filters PV solar plant, in Figure 11.

Therefore, there was no evidence of a direct relationship between the events in the external overhead line failure and disconnection in the PV solar plant.

• Impedance variation: The Z (ω) in the plant due to the sequence of events in the grid. The Z (ω) variations in the busbars of the PV solar plant, for each of the events reproduced. Therefore, the Z (ω) does not depend on the topology of the grid.

Additionally, there was no coincidence of poles and zeros with the characteristic harmonics of an electrical system (2nd, 3rd, 5th, 7th, 11th and 13th). This indicates that it is unlikely that high current and voltage demands will be placed on the PV solar plant.

**Figure 12:**
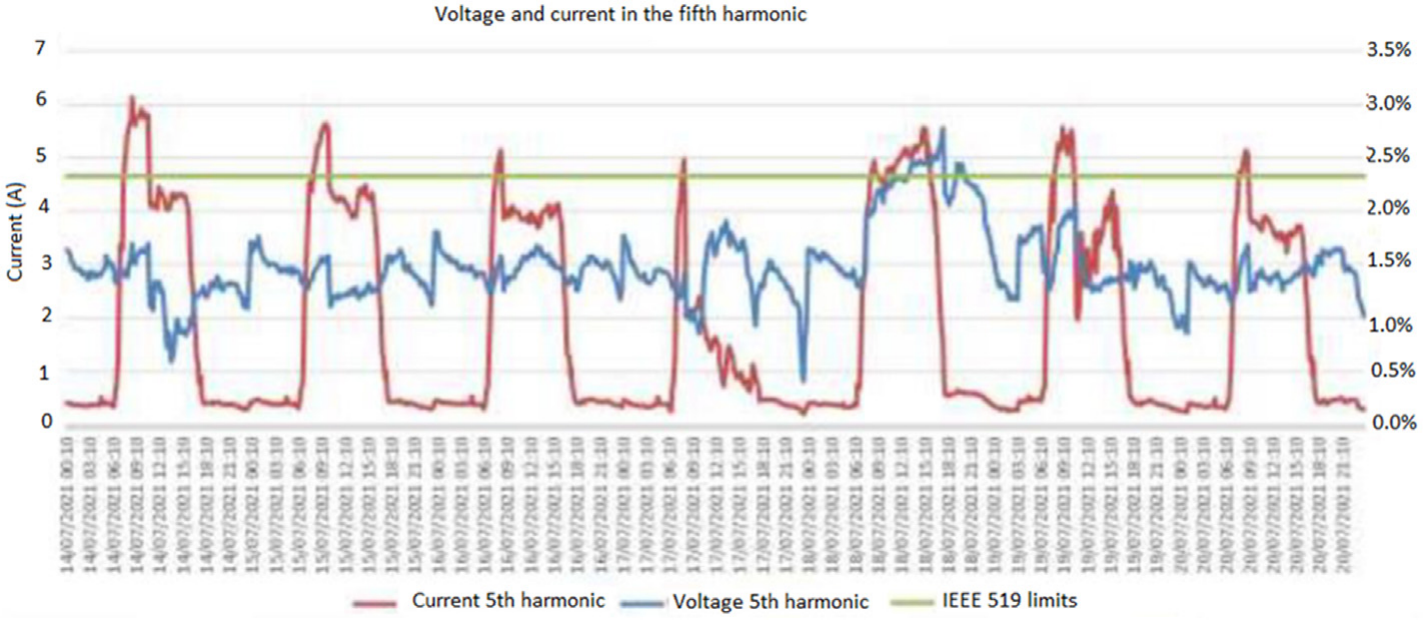
Reduction of the fundamental current.

The behavior of the 5th order harmonic current (I5) and the behavior of the fundamental current (I1) are identified in Figure 9. In the lower part of the figure, an enlargement evaluates the lower values, it is observed how, due to the control of the control unit, the fundamental current approaches 0 A in a few moments, causing all the distortions to increase to Although the harmonic current does not have a considerable variation, in Figure 12.

In all the period evaluated, the voltage distortion at the 5th harmonic is contrasted with the current distortion of the same order. It is evident that the injection of the 5th harmonic of current from the generation has a correspondence with the 5th harmonic of voltage only up to a certain period, since then the current falls and the voltage remains out of range. It is concluded that the phenomenon corresponds to some topological change made in the grid. The peaks in the morning at 6:18 and the end of the day 17:25, it creates a overload in the electrical components, especially in the AC filters associated to the DC/AC converters.

**Figure 13:**
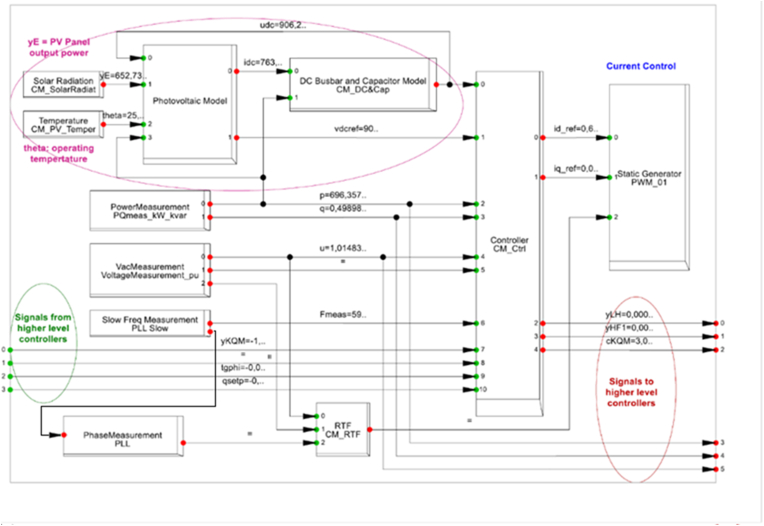
New signal incorporated in the DC/AC converter.

Besides, an important problem in the DC/AC converter, the slow frequency measurement PLL slow should give a feedback in the signal of the phase measurement PLL for the ac power action, associated to the current, voltage, irradiance and the evaluation of the grid condition, according Figure 13.

A comparison of Figure 10 and Figure 13 is a new signal from the Slow frequency measurement PLL to the Phase measurement PLL. With this new reference, the phase in slow frequency has a feedback, in order to improve the design control in the photo-voltaic converter DC/AC.

**Figure 14:**
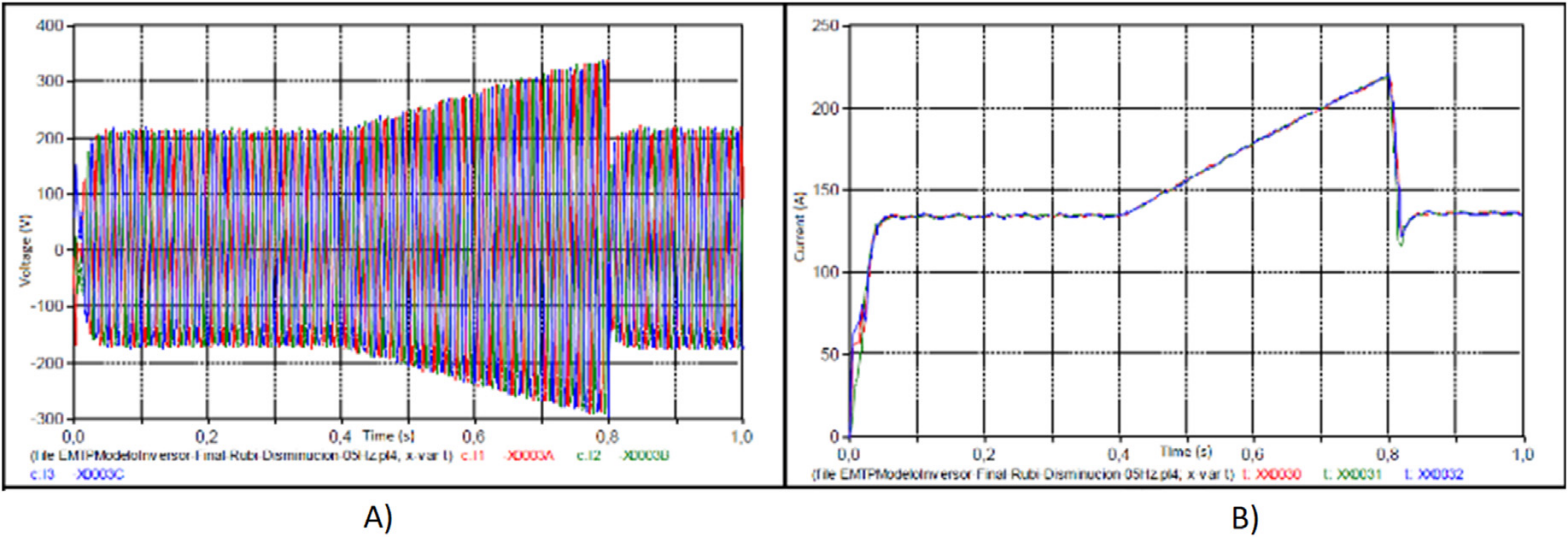
Overcurrent identified for the PLL signal, with an overcurrent: A) The voltage associated to the bad signal associated to the frequency variation, it is not detected as a normal signal by the PLL. Therefore, the current increases suddenly. B) Fundamental contribution with an overcurrent.

With the evaluation of the root cause analysis, the detection of the main failure is the bad signal programmed in the DC/AC converter, associated to the PLL devices, it caused a overcurrent in the inverter, and a sudden disconnection. The overload of the dc/ac converter caused by the overcurrent is not identified in. In Figure 14 the overcurrent is 1.62 pu caused by frequency variation of 0.3 Hz in a 60Hz grid.

**Figure 15:**
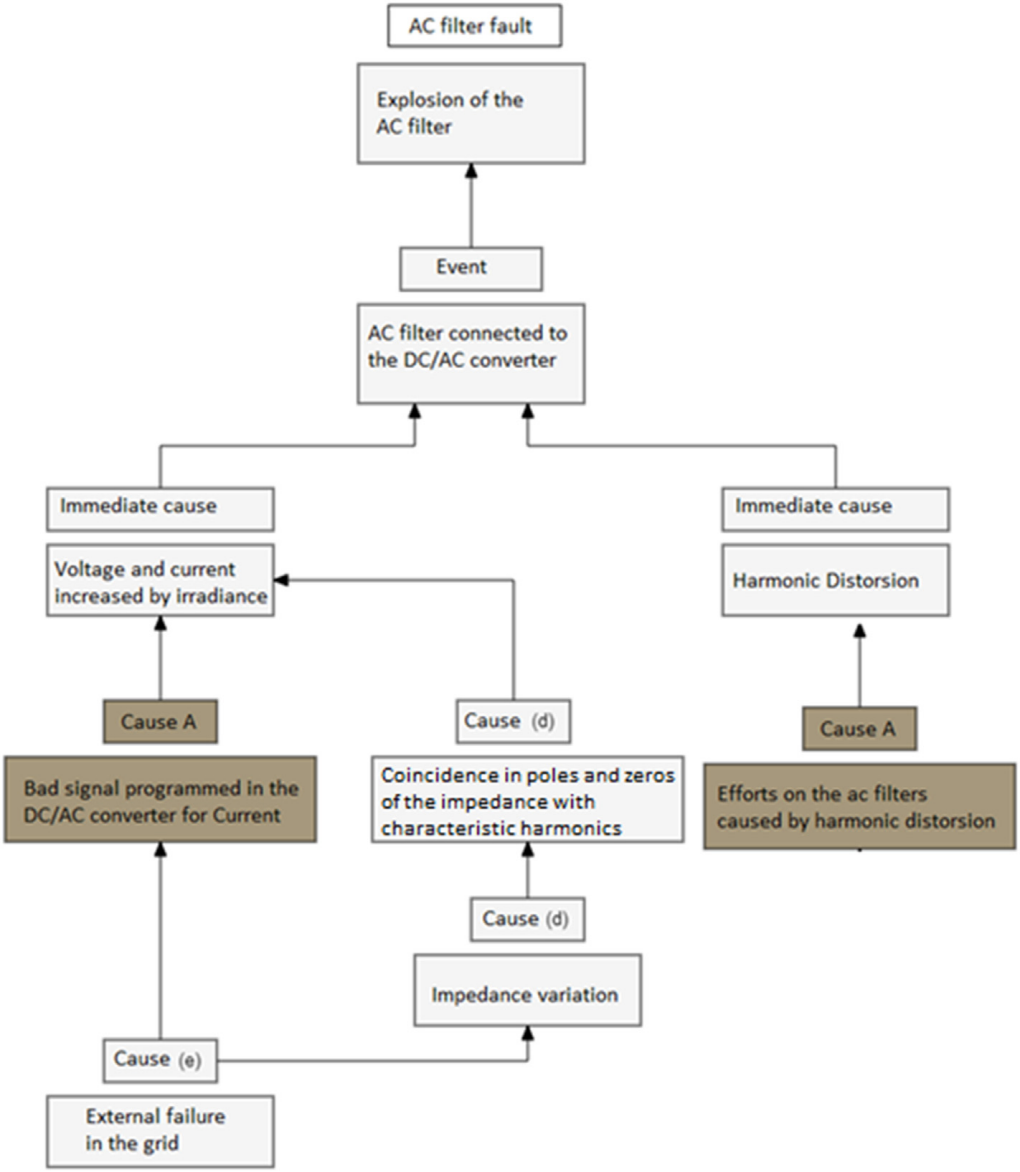
Root cause analysis associated to the main failure in the PV solar plant, ac filter fault.

**Figure 16:**
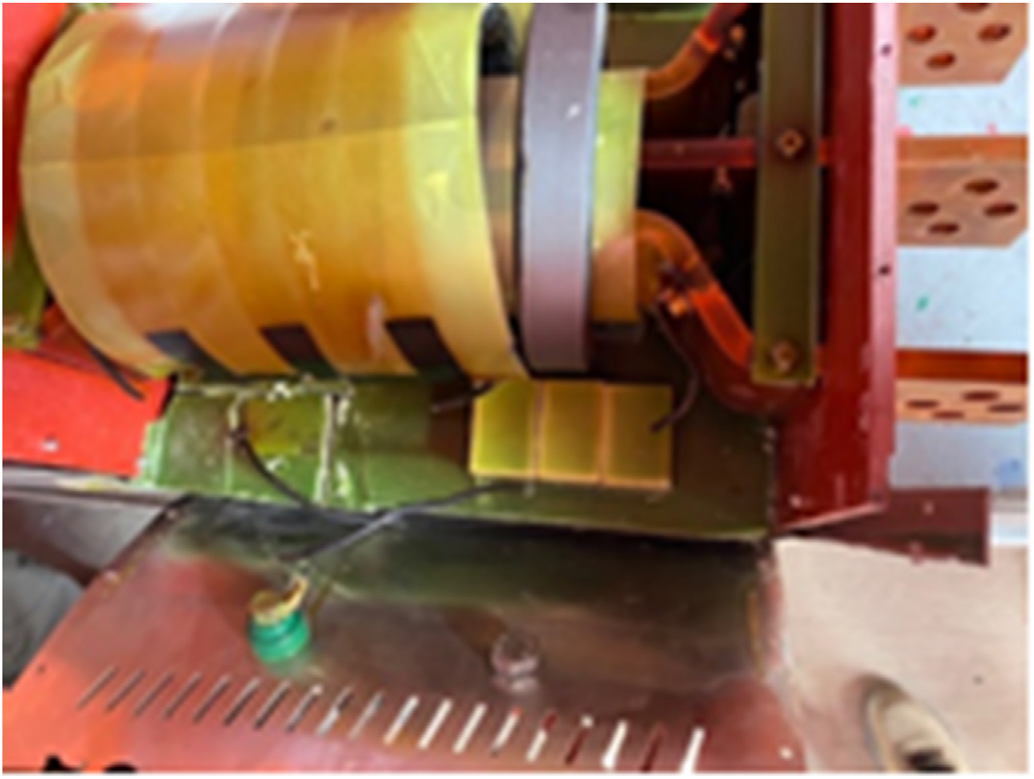
AC filter explosion.

According with the evaluation of Figure 15. The DC/AC converters in solar plants should be improved with the PLL feedback and the design should consider a over effort caused by the second harmonic in the first hour and the end of the day, these two factos caused an overcurrent in the ac filter with the explosion, Figure 16. The filter fault probability is 98.97%, according Figure 9, is detected as a Grid Fault.

On the other hand, with the knowledge managment methodology, the evaluation in the 2019 is 3.34 in the average value, and the evaluation in 2021, after of the root cause analysis is 4.69; in this case, the limit is 5.00. It demonstrated more knowledge of the engineers and a better understanding of the problem, compared with the year 2019, Figures 17 and 18.

**Figure 17:**
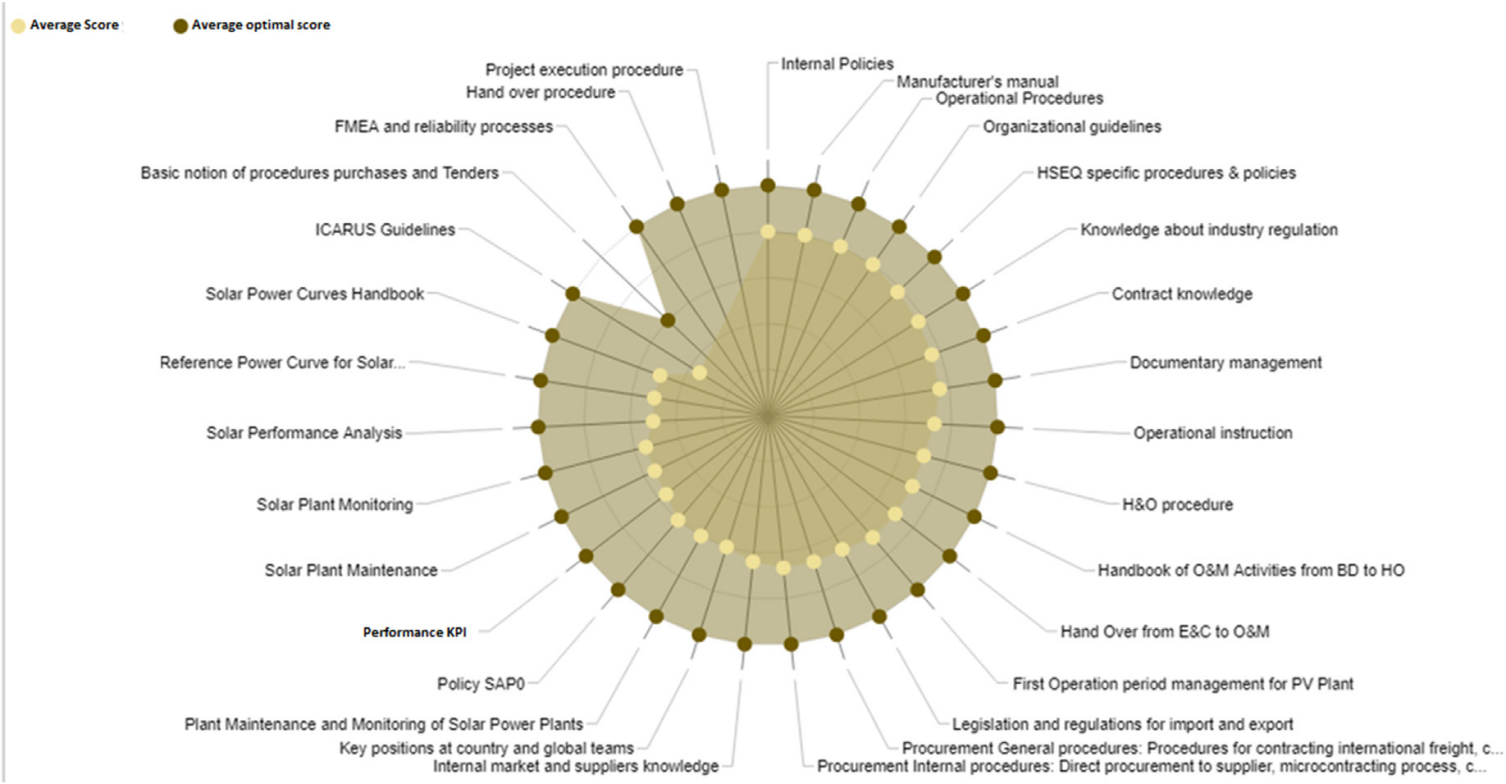
Evaluation in 2019, associated to the knowledge management.

**Figure 18:**
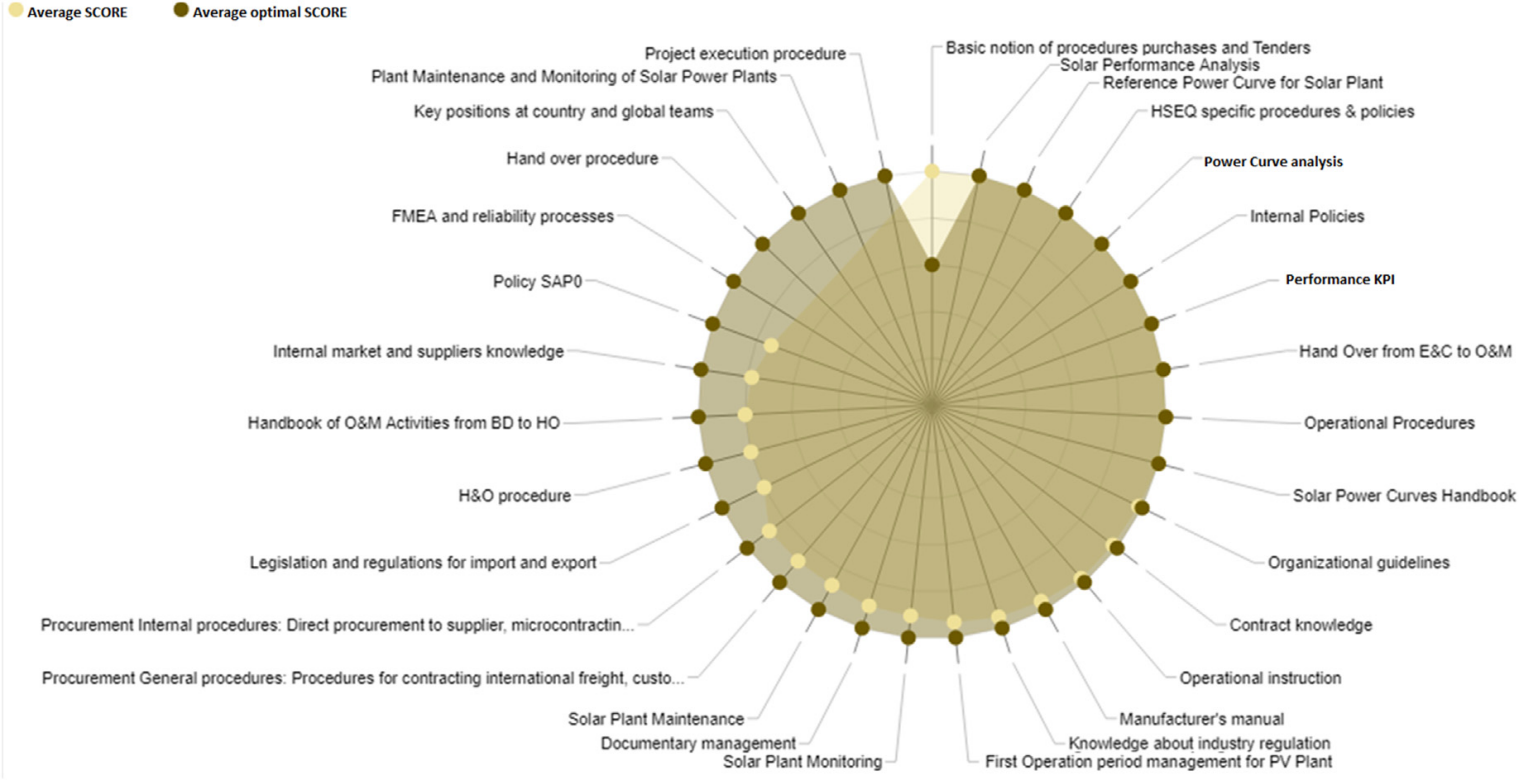
Evaluation in 2021, associated to the knowledge management.

In Figure 18, the ontology-based knowledge management methodology has been implemented for failure analysis; for example in the simulation [17], the knowledge graph has been considered for a context description [18]. In this paper, the knowledge management platform allows to incorporate the lesson learned for the failure mode, and the graphs describe the context for the inverter failure in the solar plant. Similar to other papers, “a large amount of data is required to have more accurate probabilities and a more precise knowledge retrieval model” [18].

## 4 Discussion

The evaluation of the methods applied to root cause analysis in the last years is indicated in Table 4. This research article includes the precision, recall and F-measure and node interference analysis, in this case, the precision is lower than VAR process, but the recall and F-measure are the most complete evaluation with the approach of the Erdos-Renyi model with spatial-temporal analysis for RCA associated to graph-based support vector machine.

**Table 4:** Evaluation of the precision, recall and F-measure and node interference.

Algorithms	Precision (%)	Recall (%)	F-measure (%)
Multivariate time-series data are generated using VAR process [21].	100.00	50.80	67.40
GGM with neighborhood similarity [22].	83.30	100.00	90.90
Sequential state switching and artificial anomaly association [21].	90.60	96.70	93.60
Random Committee and Logistic forest tree algorithms in a competition [3].	98.99	99.10	99.10
**New proposal:** Erdos-Renyi model with spatial-temporal analysis for RCA associated to graph-based support vector machine.	99.20	99.60	99.60

In the case study, the filter fault evaluated is the most difficult ones to be detected, according Ref. [3]. However, the rankings obtained that faults probability is 98.97% for 81.07 ​MW ​h. On the other hand, the failure as fault (0.37 ​MW ​h), soiling (0.76 ​MW ​h) or automatic actions (9.79 ​MW ​h) present low accuracy in finding, as a normal behavior of the PV solar plant.

As a summary, the Erdos-Renyi model with spatial-temporal analysis for RCA associated to graph-based support vector machine in the previous sections, it is pattern-based root-cause method with the following important contributions:

- Ability to deal with multiple nominal modes, in a spatial-temporal analysis and simultaneous failure modes with graphs methods [24].
- Highest accuracy compared with traditional spatial temporal methods for root cause analysis.
- Incorporation of the knowledge management to the data-driven approach.
- Robustness: This approach is compared with traditional RCA methods with international standards and spatial-temporal analysis; however, this new proposal has a benefit with the faulty node in both synthetic dataset and real dataset.
- Efficiency: It is validated in Table 4, with the precision, recall and F-measure.

Finally, this root-cause analysis is conducted when there is an anomaly detected and an evaluation of the conditions of the team for human error or control design with 31 factors (policy, procedure, technical knowledge, organization, solar plant monitoring, instructions, manufacturer documentation, maintenance, and contract activities) according Table 1. This new approach (data-driven plus knowledge management approach), it has mitigated the false alarms in a precision of 99.20%, in a real life. An experiment is carried out here where we perform root-cause analysis with a dataset evaluated, it has 120,561 signals associated to 3,014,025 patterns, during the period from 2018 to 2021 in a PV solar plant.

The new methodology is used for the evaluation of failures in the photo-voltaic solar plants; the application to all the renewable energy power plants is a possibility, due to configuration and topology; it is similar in wind farms, battery energy storage systems, hydroelectric power plants, hydrogen plants and thermal plants. A common way to evaluate this equipment is with the efficiency curves. Therefore, the main generator could be similar. The analysis proposed is +10 min analysis for period higher than 2 years. On the other hand, industrial equipment in mining and petroleum companies could evaluate this application for future works.

## 5 Conclusions

In this research, a new contribution for root cause analysis with the validation of a case study associated to a photo-voltaic solar plants. It develops a new methodology, to improve the quantitative analysis with the incorporation of the Erdos-Renyi model, for the failure modes and Root cause analysis. The quantitative design considers six independent variables with a failure’s mode detection, in the case study with 560880 panels and 179 MW. Furthermore, the qualitative design has considered the 31 factors for the evaluation before (2019) and after (2021). The root cause analysis has detected a bad signal programmed in the DC/AC converter, associated to the PLL devices, it caused an overcurrent in the inverter, and a sudden disconnection, sometimes with massive fuses melt during high irradiance periods [28]. The overload of the dc/ac converter caused by the overcurrent; it has a sudden frequency variation of 0.3 Hz; therefore, it was validated with the detection of the root cause, and the evaluation of the qualitative research with the improvement of the 140%. The licker scale used in the knowledge management tool (KMT) from 0 to 5; the results are the following:

In 2019, the evaluation with the KMT is 3.34; then, the analysis and the methodology implemented, the KMT is 4.69; with the application of the survey and the analysis of 31 factors, according to the information in Table 4 and Figure 2.

The evaluation of the case study was applied for three years, since 2019 to 2021; and the reduction of the production during 2018, 2019, 2020 and 2021 in Figure 9. The limitation of the method is the information available in the photo-voltaic solar plant, in this case, the minimum information is two years; and the survey was realized before and after the implementation. In consequence, this new root cause analysis for the identification of failures modes could be used for complex new business models as the generation of clean water based on PV solar plant [29] or the process for Lithium mining, it requires high reliability in theses process without sudden failures.

## Declarations

### Author contribution statement

Ricardo Manuel Arias Velásquez: Analyzed and interpreted the data; Contributed reagents, materials, analysis tools or data; Wrote the paper.

Jennifer Vanessa Mejía Lara: Conceived and designed the experiments; Performed the experiments; Analyzed and interpreted the data; Contributed reagents, materials, analysis tools or data; Wrote the paper.

### Funding statement

This research did not receive any specific grant from funding agencies in the public, commercial, or not-for-profit sectors.

### Data availability statement

The data is attached in this journal as a complementary in csv file.

### Declaration of interests statement

The authors declare no conflict of interest.

### Additional information

No additional information is available for this paper.
